# Do We Have Enough Evidence That Metformin Is Superior to Other Antidiabetic Drugs in Pancreatic Cancer Risk Reduction?

**DOI:** 10.3390/ijms27104195

**Published:** 2026-05-08

**Authors:** Izabela Szymczak-Pajor, Józef Drzewoski, Sylwia Wenclewska, Aneta Rogalska, Agnieszka Śliwińska

**Affiliations:** 1 Department of Nucleic Acid Biochemistry, Medical University of Lodz, 251 Pomorska Str., 92-213 Lodz, Poland; agnieszka.sliwinska@umed.lodz.pl; 2 Central Teaching Hospital of the Medical University of Lodz, 251 Pomorska Str., 92-213 Lodz, Poland; jozef.drzewoski@umed.lodz.pl; 3 Department of Internal Diseases and Diabetology, Medical University of Lodz, 251 Pomorska Str., 92-213 Lodz, Poland; sylwiawenclewska@wp.pl; 4 Department of Medical Biophysics, Institute of Biophysics, Faculty of Biology and Environmental Protection, University of Lodz, 141/143 Pomorska Str., 90-236 Lodz, Poland; aneta.rogalska@biol.uni.lodz.pl

**Keywords:** metformin, pancreatic cancer (PC), antihyperglycemic drugs (ADs), PC risk

## Abstract

The current literature indicates that type 2 diabetes (T2DM) significantly increases the risk of cancer, including pancreatic cancer (PC). While metformin’s primary role is the management of T2DM, its utility extends to systemic anti-cancer effects against various cancers. Nevertheless, its impact appears limited to risk reduction, as its efficacy as a primary or adjuvant treatment for established cancer remains unproven in clinical settings. This meta-analysis aimed to evaluate the association between metformin use—both as monotherapy and in combination with other antidiabetic drugs (ADs)—and the risk of PC. We synthesized data from 16 observational studies identified through PubMed, Cochrane Library, and Clinical Trials using the Population, Intervention, Comparison, Outcomes, and Study Type (PICOT) framework. The data were analyzed using Cochrane Review Manager software 5.4, with results reported as the relative risk (RR) and 95% confidence interval (95% CI) for each comparative group; statistical significance was defined as *p*-value < 0.05. Our findings indicate that metformin demonstrated a significant reduction in overall PC risk when compared to the pooled group of alternative ADs. Furthermore, metformin significantly lowers PC risk compared to sulfonylureas (SUs), alpha-glucosidase inhibitors (AGIs), and insulin. Conversely, metformin use was associated with a markedly elevated PC risk relative to thiazolidinediones (TZDs) and DPP-4 inhibitors (DPP4i). Considering metformin monotherapy vs. its combination with other ADs, we found that metformin lowered the risk of PC compared to its combination with SUs and AGIs but elevated the PC risk relative to its combination with TZDs and DPP4i. To conclude, these results suggest that metformin may protect patients with T2DM from PC development. However, individual PC risk and diabetes compliance should be taken into account when deciding whether to add an additional AD(s) to metformin therapy.

## 1. Introduction

Pancreatic cancer (PC) is a growing worldwide health challenge [1]. Its incidence is significantly higher in countries with a high human development index (HDI), particularly in North America and Europe [2]. Globally, there were over 510,000 new cases of PC and 467,000 related deaths in 2022. Moreover, it is projected to become the second leading cause of cancer-related death in the U.S. by 2030 [3]. PC continues to have one of the poorest prognoses among all cancers, largely due to late-stage diagnosis [4]. While the 5-year relative survival rate in the U.S. has shown a slight but steady upward trend, reaching 13%, the global 5-year survival rate remains approximately 10% [5,6].

The diagnosis of PC is frequently delayed because the disease is typically asymptomatic or presents only non-specific symptoms, such as unexplained weight loss or back pain, in its early stages. Consequently, the majority of cases are identified only after the cancer has progressed to a locally advanced or metastatic stage, severely restricting treatment options [7,8]. Surgical resection remains the only treatment with curative potential, yet only 15–20% of patients are eligible for surgery at the time of diagnosis [7]. Recent research highlights the pivotal role of the tumor microenvironment (TME) in driving the disease’s high aggressiveness, invasiveness, and resistance to therapy. Specifically, the TME, which is defined as a dense network of non-cancerous cells and proteins known as the stroma, often constitutes the majority of the tumor mass and is a major determinant of this aggressive phenotype [9].

While the median age at diagnosis remains around 70, there is a noted increasing burden among younger adults, particularly women [1,6]. Non-hereditary, modifiable risk factors include smoking, obesity, ethanol consumption, chronic pancreatitis, metabolic syndrome, and type 2 diabetes mellitus (T2DM) [10]. T2DM represents a rapidly escalating global health crisis, primarily driven by demographic shifts, urbanization, and lifestyle changes, accounting for over 90% of all diabetes cases worldwide [11,12]. Extensive epidemiological, observational, and mechanistic data confirm that T2DM is an independent risk factor associated with an increased incidence and mortality across a variety of cancers [13,14,15]. The increased cancer risk observed in patients with T2DM stems from metabolic and hormonal disturbances that generate a favorable microenvironment for tumor initiation and progression [13,14]. This link is mediated by several common biological pathways, most notably: hyperinsulinemia and the insulin growth factor (IGF) axis, chronic inflammation, hyperglycemia, and oxidative stress [13,16,17,18,19,20]. A striking finding in PC epidemiology is that nearly 80% of PC patients exhibit either new-onset T2DM or impaired glucose tolerance at the time of initial diagnosis [21,22].

The association between T2DM and PC has garnered significant scientific attention. While the precise relationship between diabetes duration and the magnitude of PC risk remains a subject of clinical debate, there is a near-universal consensus that patients with T2DM face a significantly elevated risk of developing PC [23,24,25]. Consequently, clarifying how specific anti-diabetic medications modulate the incidence of PC has emerged as a critical area of investigation. According to the current Standards of Care in Diabetes 2026, treating T2DM with metformin monotherapy or metformin in combination with one other AD is the first-line therapy in patients with T2DM without other comorbidities or risks. In turn, for individuals with T2DM and confirmed disease or high-risk indicators of atherosclerotic cardiovascular disease, heart failure, or chronic kidney disease, it is recommended to use an SGLT2 inhibitor and/or GLP-1RA with proven cardiovascular benefits. This approach is advised regardless of the HbA1c level, with or without metformin, while taking into account patient-specific factors [26].

Metformin is widely recognized for its potential to favorably impact the prognosis of cancer patients [27,28]. The drug’s anticancer properties are well-documented in both in vitro and observational studies. Its primary mechanisms include AMPK pathway activation and the blockage of mitogen signaling. Metformin activates the LKB1/AMPK/mTOR signaling pathway. The activation of AMPK suppresses the mTOR pathway, a critical regulator of cell growth and proliferation. Additionally, metformin reduces circulating insulin levels, thereby inhibiting insulin-induced tumor growth [29,30]. A significant body of pre-clinical evidence supports these mechanisms. In vitro studies have demonstrated that metformin inhibits cancer cell proliferation, migration, and invasion, while exhibiting a selective cytotoxic effect against cancer stem cells [31,32]. These encouraging findings have been further corroborated in in vivo studies [33].

Beyond pre-clinical investigations, the clinical potential of metformin in oncology is being actively assessed. Several clinical trials (e.g., NCT01167738 and NCT01210911) have been designed to evaluate the efficacy of metformin in combination with standard chemotherapy regimens for PC. However, data from these clinical trials indicate that metformin may lack therapeutic efficacy in the management of established PC. In contrast, epidemiological studies have consistently linked metformin use to a reduced overall cancer risk in patients with T2DM [34,35]. Specifically, evidence suggests that in patients diagnosed with PC, treatment regimens including metformin are associated with a significant increase in overall survival and the 5-year survival rates compared to those utilizing alternative ADs or no treatment [36,37]. However, the question of whether metformin use influences the incidence rate (risk of development) of PC remains inconsistently addressed across the current body of literature. To address this critical knowledge gap, we conducted a rigorous meta-analysis to definitively clarify the association between metformin utilization in T2DM patients and the risk of developing PC.

## 2. Results

### 2.1. The Studies Included in the Meta-Analysis

**Figure 1:**
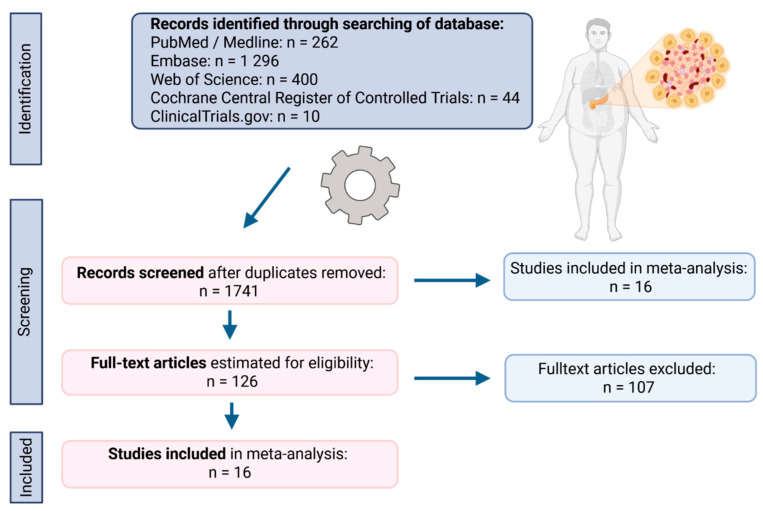
A flowchart of the screening procedure.

As illustrated in Figure 1 (flow diagram), the initial database search yielded 2012 records. Following the removal of 271 duplicates, the remaining articles underwent a rigorous screening process. After a comprehensive full-text evaluation and quality assessment, 16 articles met the predefined inclusion criteria and were incorporated in the meta-analysis.

### 2.2. Metformin Use Mitigates the Risk of PC Compared to the Pooled Group of Other ADs

**Figure 2:**
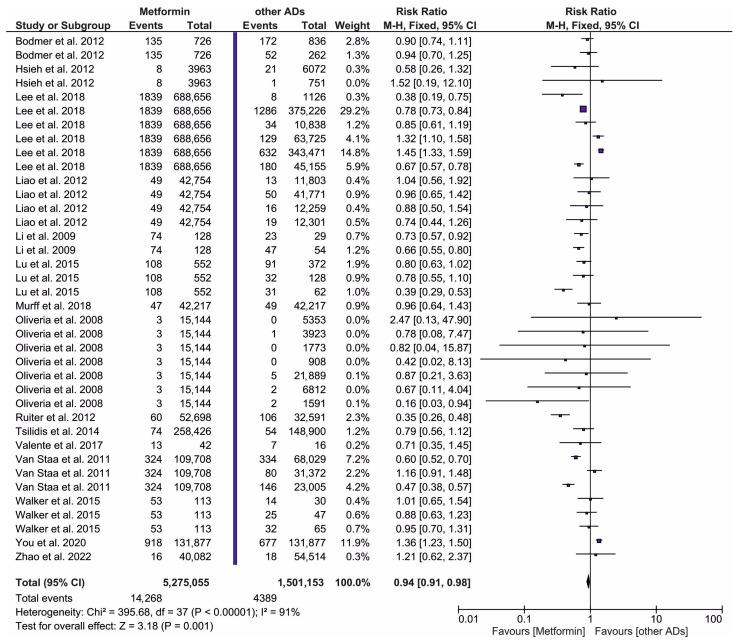
The risk ratio for the incidence of PC in patients taking metformin (intervention) vs. the pooled group of antidiabetic drugs other than metformin (ADs (comparator)) [38,39,40,41,42,43,44,45,46,47,48,49,50,51,52].

The risk ratio of PC in T2DM patients utilizing metformin (intervention) in comparison to those receiving ADs other than metformin (comparator) is depicted in Figure 2. A total of sixteen original publications were deemed eligible for inclusion in this meta-analysis. The pooled dataset included 5,275,055 patients on metformin (intervention group) and 1,501,153 patients in the pooled group, including users of Ads other than metformin. The meta-analysis results demonstrated a pronouncedly reduced risk of PC (RR = 0.94; 95% CI: 0.91, 0.98, *p* = 0.001) in metformin users compared to patients treated with other than metformin ADs.

### 2.3. Metformin Monotherapy Reduced Risk of PC When Compared to Monotherapy with SUs, AGIs, Meglitinides, and Insulin, Yet Was Associated with an Elevated Risk Relative to TZDs and DPP4i

**Figure 3:**
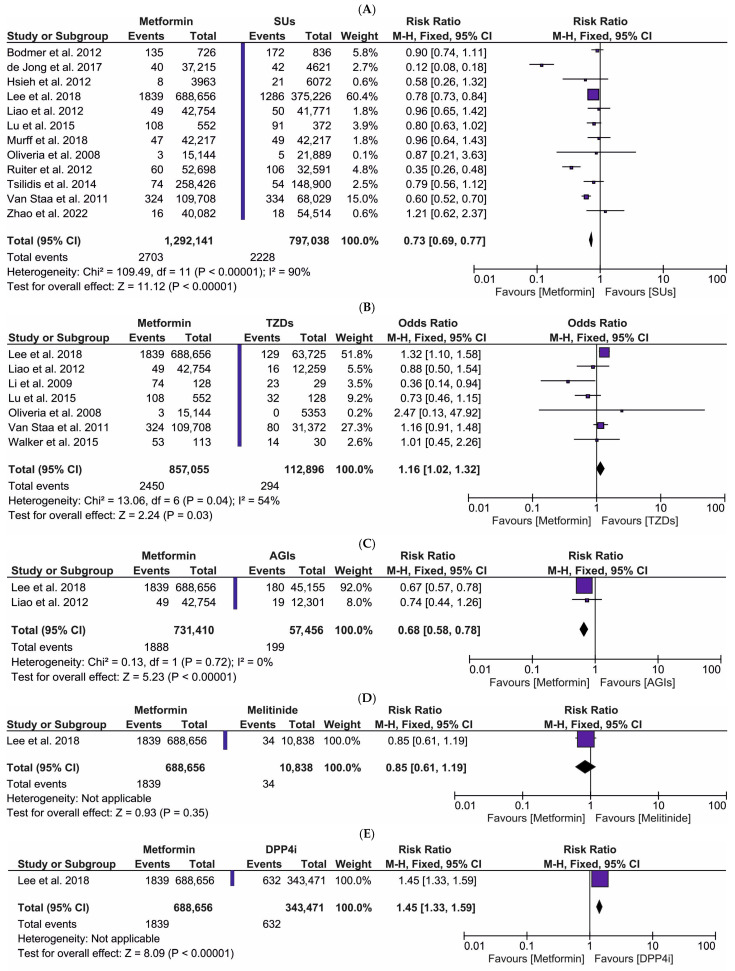

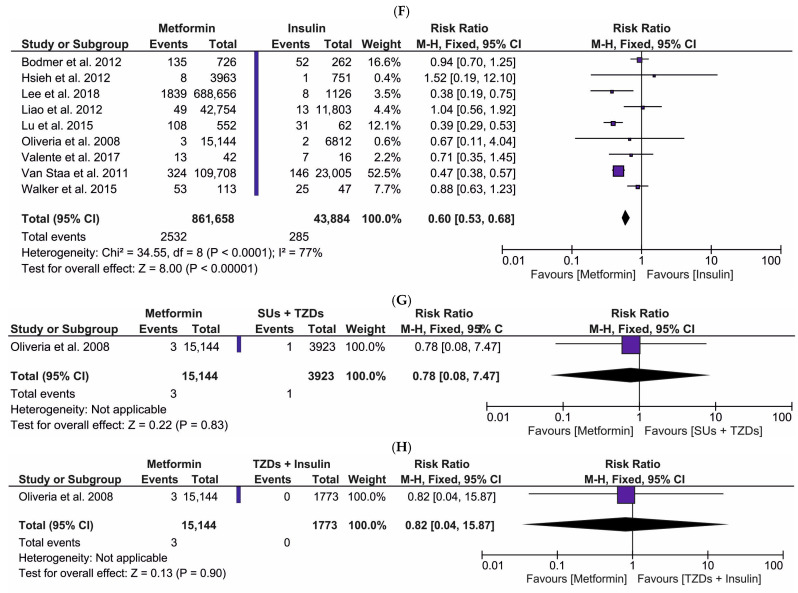
The risk ratio for the incidence of PC in patients taking metformin (intervention) vs. (**A**) sulfonylureas (SUs); (**B**) thiazolidinediones (TZDs); (**C**) alpha-glucosidase inhibitors (AGIs); (**D**) meglitinides; (**E**) dipeptidyl peptidase 4 inhibitors (DPP4i); (**F**) insulin; (**G**) SUs + TZDs; and (**H**) TZDs + insulin (comparator) [38,39,40,41,42,43,44,45,46,48,49,50,51,52,53].

The forest plot illustrating the risk ratio of PC in patients utilizing metformin (intervention) vs. various ADs (comparator): (A) SUs, (B) TZDs, (C) AGIs, (D) meglitinides, (E) DPP4i, (F) insulin, (G) SUs combined with TZDs, and (H) TZDs combined with insulin is shown in Figure 3. For the comparison of metformin vs. SUs, TZDs, AGIs, meglitinides, DPP4i, insulin, SUs combined with TZDs, and TZDs combined with insulin, a total of 15 original publications were eligible for inclusion in the meta-analysis. The pooled data included: (A) 1,292,141 metformin users (intervention group) vs. 797,038 users of SUs (comparative group); (B) 856,503 patients on metformin (intervention group) vs. 112,768 patients on TZDs (comparative group); (C) 731,410 metformin users (intervention group) vs. 57,456 users of AGIs (comparative group); (D) 688,656 patients taking metformin (intervention group) vs. 10,838 patients receiving meglitinides (comparative group); (E) 688,656 metformin users (intervention group) vs. 343,471 users of DPP4i (comparative group); (F) 552 patients utilizing metformin (intervention group) vs. 128 patients on insulin (comparative group); (G) 15,144 patients treated with metformin (intervention group) vs. 3923 patients on SUs and TZDs (comparative group); and (H) 15,144 patients taking metformin (intervention group) vs. 1773 patients receiving TZDs combined with insulin (comparative group). The meta-analysis showed a markedly decreased risk of PC (RR = 0.73; 95% CI: 0.69, 0.77, *p* < 0.00001) in patients receiving metformin relative to those taking SUs (Figure 3A). Similarly, we observed a pronouncedly reduced risk of PC (RR = 0.68; 95% CI: 0.58, 0.78, *p* < 0.00001) in metformin users compared to users of AGIs (Figure 3C). Our meta-analysis also demonstrated a significantly lower risk of PC (RR = 0.60; 95% CI: 0.53, 0.68, *p* < 0.00001) in patients treated with metformin than patients on insulin (Figure 3F). Interestingly, we found a markedly increased risk of PC (RR = 1.16; 95% CI: 1.02, 1.32, *p* < 0.03) in the metformin intervention group relative to the TZDs group (Figure 3B). Moreover, the meta-analysis also revealed a pronouncedly elevated risk of PC (RR = 1.45; 95% CI: 1.33, 1.59, *p* < 0.00001) in the group of patients utilizing metformin compared to those taking DPP4i (Figure 3E). We did not observe any statistically significant risk of PC (RR = 0.85; 95% CI: 0.61, 1.19, *p* = 0.35) in patients on metformin compared to those treated with meglitinides (Figure 3D). Furthermore, we found no pronounced risk of PC (RR = 0.78; 95% CI: 0.08, 7.47, *p* = 0.83) in metformin users compared to those receiving SUs combined with TZDs (Figure 3G). Lastly, this meta-analysis did not reveal any significant risk of PC (RR = 0.82; 95% CI: 0.04, 15.87, *p* = 0.90) in the group of patients treated with metformin compared to users of TZDs and insulin (Figure 3H). In summation, these results indicate that metformin monotherapy confers a lower PC risk when compared to SUs, AGIs, and insulin monotherapies, but an elevated PC risk when compared to TZDs and DPP4i monotherapies.

### 2.4. The Use of Metformin in Monotherapy Did Not Affect the Risk of PC Relative to the Metformin Combined with Other ADs

**Figure 4:**
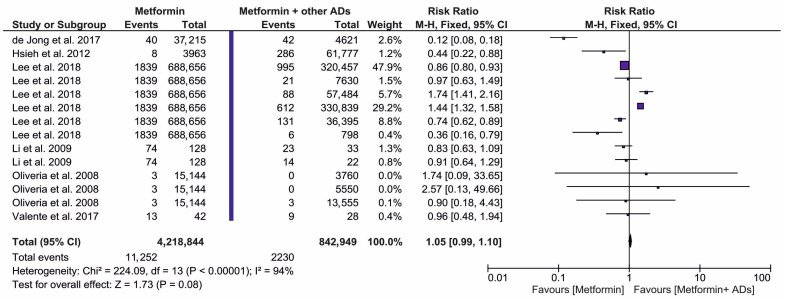
The risk ratio for the incidence of PC in metformin users (intervention) vs. users of combined treatment including metformin and other antidiabetic drugs (ADs (comparator)) [39,40,43,49,51,53].

Figure 4 presents the risk ratio of PC in patients who were treated only with metformin (intervention) vs. those taking metformin combined with other ADs (comparator). A total of six original publications were eligible for inclusion in this meta-analysis. The pooled dataset included 4,218,844 patients in the metformin intervention group and 842,949 patients in the metformin combined with other ADs comparative group. The meta-analysis results did not find any statistically significant risk of PC (RR = 1.05; 95% CI: 0.99, 1.10, *p* = 0.08) in the group of patients on metformin compared to those treated with metformin and other ADs. Interestingly, these findings reveal a similar PC risk across treatment regimens comprising metformin alone and metformin administered in combination with other ADs.

### 2.5. Metformin Monotherapy Showed a Lower Risk of PC than Metformin Combined with SUs or AGIs, but a Higher Risk Relative to Its Combinations with TZDs or DPP4i

**Figure 5:**
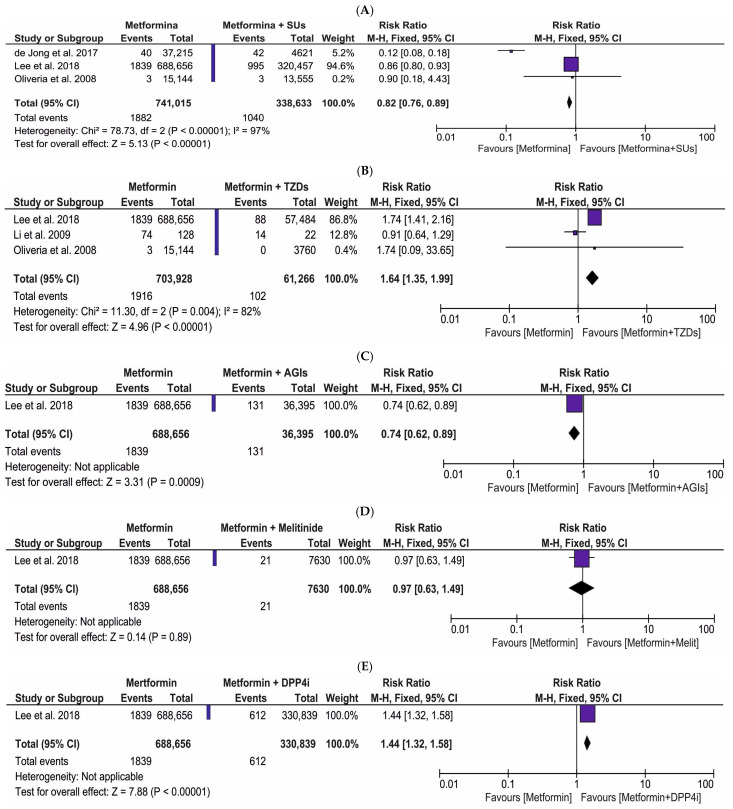

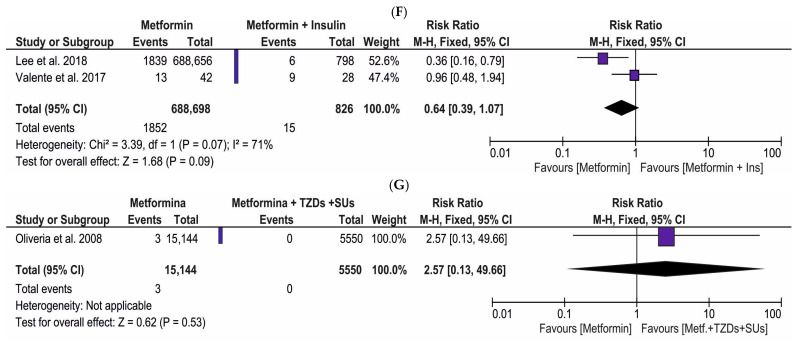
The risk ratio for the incidence of PC in T2DM patients on metformin monotherapy (intervention) vs. combined therapy (comparator) composed of metformin with (**A**) sulfonylureas (SUs); (**B**) thiazolidinediones (TZDs); (**C**) alpha-glucosidase inhibitors (AGIs); (**D**) meglitinides; (**E**) dipeptidyl peptidase 4 inhibitors (DPP4i); (**F**) insulin; and (**G**) TZDs and SUs [40,43,49,51,53].

Figure 5 presents a comprehensive forest plot illustrating the risk ratio of PC in patients utilizing metformin monotherapy (intervention) vs. regimens combining metformin with various ADs (comparator): (A) SUs, (B) TZDs, (C) AGIs, (D) meglitinides, (E) DPP4i, (F) insulin, and (G) SUs and TZDs. For these comparisons, a total of five original publications were eligible for inclusion in the meta-analysis. The pooled data included: (A) 741,015 metformin users (intervention group) vs. 338,633 users of metformin and SUs (comparative group); (B) 703,928 patients on metformin (intervention group) vs. 61,266 patients on metformin combined with TZDs (comparative group); (C) 688,656 patients treated with metformin (intervention group) vs. 36,395 patients taking metformin and AGIs (comparative group); (D) 688,656 patients utilizing metformin (intervention group) vs. 7630 patients receiving metformin combined with meglitinides (comparative group); (E) 688,656 metformin users (intervention group) vs. 330,839 users of metformin and DPP4i (comparative group); (F) 688,656 patients taking metformin (intervention group) vs. 826 patients on metformin combined with insulin (comparative group); and (G) 15,144 patients receiving metformin (intervention group) vs. 5550 patients utilizing metformin combined with SUs and TZDs (comparative group). The meta-analysis showed a significantly reduced risk of PC (RR = 0.82; 95% CI: 0.76, 0.89, *p* < 0.00001) in metformin users relative to patients receiving metformin combined with SUs (Figure 5A). Similarly, we found a markedly decreased risk of PC (RR = 0.74; 95% CI: 0.62, 0.89, *p* < 0.0009) in patients on metformin monotherapy compared to those treated with metformin and AGIs (Figure 5C). Interestingly, our meta-analysis revealed a pronouncedly higher risk of PC (RR = 1.64; 95% CI: 1.35, 1.99, *p* < 0.00001) in patients utilizing metformin than those taking metformin combined with TZDs (Figure 5B). We also observed a significantly elevated risk of PC (RR = 1.44; 95% CI: 1.32, 1.58, *p* < 0.00001) in the metformin intervention group relative to metformin and DPP4i users (Figure 5E). The meta-analysis did not demonstrate any statistically significant risk of PC (RR = 0.97; 95% CI: 0.63, 1.49, *p* < 0.89) in the group of metformin users compared to those receiving metformin combined with meglitinides (Figure 5D). Furthermore, we did not observe any marked risk of PC (RR = 0.64; 95% CI: 0.39, 1.07, *p* = 0.09) in the patients on metformin compared to those treated with metformin and insulin (Figure 5F). Lastly, our meta-analysis also did not identify any pronounced risk of PC (RR = 2.57; 95% CI: 0.13, 49.66, *p* = 0.53) in users of metformin monotherapy relative to those taking metformin combined with TZDs and SUs (Figure 5G). Collectively, metformin monotherapy demonstrated a lower relative PC risk when compared to its combination with SUs or AGIs but an elevated risk when compared to its combination with TZDs or DPP4i.

## 3. Discussion

It is well recognized that T2DM is associated with an elevated risk of several cancers, particularly liver cancer and PC [54]. Interestingly, recent evidence suggests that the risk of PC is significantly higher in the initial two years following a T2DM diagnosis. Compensatory hyperinsulinemia, a hallmark of nascent T2DM, likely drives a mitogenic response that promotes the rapid progression of pre-malignant precursors into overt PC [55,56]. Metformin, the most widely prescribed anti-diabetic agent, is recognized for its anti-cancer properties, and its potential role as an adjuvant in cancer combination therapy has been proposed [57,58]. The anti-cancer effect of metformin is intimately linked to its glucose-lowering capacity mediated through multiple pathways. Firstly, the drug improves hepatic insulin resistance, thereby lowering endogenous glucose output, primarily via the suppression of gluconeogenesis [59]. Secondly, metformin increases insulin-stimulated glucose uptake in skeletal muscle, concomitant with elevated AMPK activity and phosphorylation [60,61]. Thirdly, it involves the modulation of the intestinal microbiome composition, which further enhances its glucose-lowering effect, the alteration of hormone secretion (notably glucagon-like peptide-1 (GLP-1) and the growth and differentiation factor 15 (GDF15)), the modification of erythrocyte glucose metabolism, and delayed gastric emptying [62,63]. Therefore, metformin therapy may be associated with a reduction in the risk of cancer due to its antihyperglycemic effect.

Moreover, metformin has been shown to inhibit the NF-kB signaling pathway, reducing the production of pro-inflammatory cytokines. Chronic inflammation is a well-known driver of carcinogenesis, including PC. It was found that metformin reprograms the immune system to better recognize and attack early cancer cells [64,65,66]. In contrast to metformin, exogenous insulin therapy may provide a pro-growth stimulus as insulin is a potent mitogen. When administered exogenously, it binds to IR and cross-reacts with IGF-1R and those located on pancreatic ductal cells. This binding triggers two critical oncogenic cascades: the PI3K/Akt/mTOR pathway, which promotes cell survival and inhibits apoptosis, and the Ras/MAPK/ERK pathway, which drives rapid cellular proliferation [67,68,69,70].

Among studies investigating metformin and PC, research focusing on patient survival rate and overall survival predominates; these studies demonstrate that diabetic patients diagnosed with PC derive a clinical benefit from metformin use [71,72]. Furthermore, the compelling literature suggests that metformin should be considered the first-line pharmacological agent for managing T2DM in cancer patients. Compared to alternative ADs, metformin use is associated with a reduced risk of cancer-related mortality. This protective effect is particularly pronounced for cancers such as colorectal cancer and PC [73]. However, the specific association between the use of metformin (alone or in combination with other ADs) and the risk of PC remains not yet fully elucidated, especially when compared directly to alternative AD regimens. Therefore, this meta-analysis was designed to provide a comprehensive evaluation and clarification of the interplay between metformin utilization (alone or in combination with other ADs) and the risk of PC.

Clinical evidence suggests that metformin administration is related to improved prognostic outcomes in patients with comorbid PC and diabetes, such as increased overall survival and prolonged progression-free survival. A retrospective cohort study performed by Sadeghi et al., involving 302 diabetic patients with PC, demonstrated a significant survival advantage for metformin users. The 2-year survival rate was notably higher in the metformin-treated group (30.1%) compared to the (15.4%) non-metformin cohort group treated with ADs other than metformin or any ADs (*p* = 0.004). After adjusting for potential confounders, metformin utilization was associated with a 32% reduction in the risk of mortality, highlighting its potential therapeutic benefit in improving overall survival within this patient population [74].

The efficacy of metformin in the chemoprevention and therapeutic management of PC is further substantiated by robust in vitro and in vivo experimental evidence. In vitro investigations have demonstrated that metformin significantly attenuates the proliferative capacity, colony-forming potential, and metastatic potential (including migration and invasion) of various PC cell lines (i.e., AsPC-1, BxPC-3, PANC-1, and MIAPaCa-2) [75,76]. Additionally, metformin induces programmed cell death (apoptosis) and triggers cell cycle arrest, effectively disrupting the oncogenic progression of PC cells l [77]. Evidence from in vivo studies utilizing nude xenograft models further corroborates these findings, demonstrating that metformin administration significantly inhibits the tumor growth of PANC-1 and MIAPaCa-2 adenocarcinoma [33]. Mechanistically, Kourelis et al. elucidated that metformin-mediated cancer growth suppression is driven by the activation of the LKB1/AMPK axis [78]. The activation of this metabolic sensor subsequently antagonizes the mammalian target of rapamycin (mTOR), a critical regulator of protein synthesis, cellular proliferation, and oncogenic signaling [78]. Furthermore, emerging evidence identifies the modulation of microRNAs (miRNAs), specifically miR-26a, as a novel and critical mechanism through which metformin exerts its potent anti-proliferative and pro-apoptotic effects [79]. Beyond its direct cellular impact, metformin significantly reduces circulating insulin levels, thereby antagonizing the insulin receptor/insulin-like growth factor I receptor (IR/IGF-IR) axis, a pathway frequently implicated in PC oncogenesis [76]. Collectively, these systemic and molecular actions contribute to a robust inhibition of carcinogenesis and tumor progression. Some studies have expanded these findings, demonstrating that metformin can effectively reduce the population of PC stem cells and attenuate their functional properties, such as self-renewal and chemoresistance [32,80]. In addition to its molecular and systemic actions, several investigations suggest that metformin acts as a radiosensitizer and chemosensitizer, enhancing the efficacy of standard radiotherapy [81,82] and chemotherapy [83,84]. In hamsters fed with a high-fat diet, metformin administered via drinking water prevented PC carcinogenesis induced by N-nitrosobis (2-oxopropyl) amine [85]. In diet-induced obese/pre-diabetic mice, the drug lowered PC tumor growth and downregulated signaling related to mTOR [86]. In KC (LSL-Kras^G12D/ + ^; p48-Cre), metformin effectively prevented weight gain, liver steatosis, hyperlipoproteinemia, and hyperinsulinemia in mice exposed to a high-fat, high-calorie diet. Furthermore, it halted the progression of late-stage pancreatic intraepithelial neoplasia (PanIN) lesions and the development of KRAS-driven pancreatic ductal adenocarcinoma (PDAC) promoted by diet-induced obesity [87]. Other studies have shown that oral metformin significantly alters the duodenal regional microbiome and suppresses the development of PanIN lesions in diet-induced obesity models [88]. Metformin intake delayed the occurrence of pancreatic tumors in KC mouse models, reducing the percentage of both early-stage and late-stage mPanIN lesions (mPanIN2 and mPanIN3) [89]. Despite these promising findings, rigorous clinical investigations remain necessary to definitively confirm metformin’s anti-cancer utility and further elucidate the complex molecular landscape underlying its activities.

We identified that metformin monotherapy was associated with a lower relative risk of PC incidence compared to treatment regimens involving a pooled group of other than metformin ADs. Consistent with our findings, a meta-analysis conducted by Wang et al. demonstrated that metformin treatment was associated with a 37% reduction in PC risk compared to other anti-diabetic treatment modalities (RR = 0.63; 95% CI: 0.46; 0.86; *p* < 0.05) [90]. Furthermore, a meta-analysis conducted by Hu et al. also identified that metformin use is associated with a significantly reduced risk of PC (0.82; 95% CI: 0.69; 0.98) [91]. Several earlier meta-analyses have similarly identified metformin as a protective factor against PC, reporting significant risk reductions of 36%, 37%, and 46% [90,92,93]. In turn, Singh et al. reported no significant association between metformin therapy and PC risk [94]. A recent comprehensive meta-analysis [95] investigating the relationship between metformin and the total cancer incidence corroborated that metformin utilization is associated with a diminished risk of PC. When the analysis was stratified by the control group composition (never-users or alternative ADs users), the protective effect of metformin remained significant compared to both never-users (OR  =  0.62; 95% CI: 0.45; 0.84) and those receiving alternative ADs (OR  =  0.57; 95% CI: 0.35; 0.93).

This meta-analysis demonstrated that metformin monotherapy was associated with a similar risk of PC compared to the pooled treatment regimens involving metformin combined with other ADs. Our results also showed that metformin monotherapy confers a lower PC risk relative to monotherapies utilizing SUs, AGIs, and insulin. Conversely, metformin monotherapy exhibited an elevated PC risk when analyzed against monotherapies involving TZDs or DPP4i. The disparity in PC risk between metformin and SUs is driven by fundamentally different effects on insulin signaling and cellular energy metabolism. Metformin’s lower risk profile is due to its ability to inhibit oncogenic pathways through the activation of AMPK [96]. Metformin reduces hepatic glucose production and improves peripheral insulin sensitivity. This leads to a systemic reduction in circulating insulin and insulin growth factor-1 (IGF-1) levels. Since insulin is a potent mitogen, lowering its concentration starves potential tumor cells of growth signals [97,98]. Within the cell, metformin inhibits complex I of the mitochondrial respiratory chain. This increases the AMP/ATP ratio, activating AMPK. Activating AMPK leads to the inhibition of the mTOR pathway, which is the primary regulator of protein synthesis and cell proliferation [99]. In contrast, SUs stimulate the tissue environment that can promote tumor growth as they are insulin secretagogues. SUs work by closing K_ATP_ channels on pancreatic β-cells, forcing the release of endogenous insulin. This results in local hyperinsulinemia within the pancreatic microenvironment [100]. High local insulin concentrations stimulate its binding to an insulin receptor (IR) and IFG-1 receptors (IGF-1R) on pancreatic ductal cells. This activates the PI3K/AKT and Ras/AMPK pathways—two of the most critical signaling cascades involved in the development of PC [67,68,101].

We observed that metformin monotherapy was associated with a reduced risk of PC compared to AGIs monotherapy and metformin combined with AGIs therapy. AGIs work purely locally in the small intestine. AGIs target only postprandial (after meal) insulin spikes by slowing carbohydrate absorption. While this is beneficial, it does not address fasting hyperinsulinemia or improve systemic insulin sensitivity as effectively as metformin [102].

Both metformin and TZDs are generally considered protective agents against cancer [103,104]. The results of our meta-analysis have shown that metformin elevated PC risk compared to TZDs monotherapy or metformin combined with TZDs therapy. Thus, the results presented a higher PC risk or a lower protective effect of metformin than TZDs. The efficacy of TZDs may be attributed to their peripheral insulin and sensitizing effects, which mitigate systemic hyperinsulinemia and enhance peripheral insulin sensitivity. Furthermore, the pleiotropic effects of TZDs, including their anti-inflammatory and antioxidant properties, likely contribute to this protection, as chronic inflammation and insulin resistance are established drivers of oncogenesis [105,106,107].

We observed that metformin increased PC risk compared to DPP4i monotherapy or metformin combined with DPP4i therapy. While metformin focuses on systemic energy restriction, DPP-4i targets the pancreatic microenvironment more specifically through the glucagon-like peptide-1 (GLP-1) axis. Recent evidence indicates that the use of GLP-1 receptor agonists is associated with a markedly reduced incidence of PC in patients with pancreatitis, as well as those with comorbid pancreatitis and T2DM. DPP-4i works by preventing the breakdown of GLP-1. Some studies suggest that sustained, physiological levels of GLP-1 actually protect pancreatic tissue [108,109,110,111]. GLP-1 receptor activation in the pancreas has been shown to reduce oxidative stress and apoptosis of β-cells. By maintaining a stable pancreatic environment, DPP-4i are suggested to prevent the chronic low-grade inflammation that triggers the transition from pre-cancerous to invasive cancer [112,113,114]. It was observed that DPP-4i (sitagliptin) can directly reduce the production of pro-inflammatory cytokines like IL-6 and MCP-1 within the pancreatic tissue [115].

Taken together, while metformin’s systemic AMPK activation is beneficial for overall metabolic health, the unique pancreatic tumor microenvironment (TME) may be more sensitive to the local pathways targeted by TZDs and DPP4i. The statistical superiority of DPP4i (such as sitagliptin) may stem from its direct impact on the localized inflammation. By maintaining physiological levels of GLP-1, these agents have been shown to downregulate the production of pro-inflammatory cytokines such as IL-6 and MCP-1 within the pancreatic tissue. Notably, IL-6 is a known driver of the epithelial-to-mesenchymal transition (EMT) in pancreatic ductal cells [116]. Similarly, TZDs act as high-affinity ligands for PPAR-γ, which is highly expressed in pancreatic stellate cells. Activation of PPAR-γ can revert these cells to a quiescent state, thereby inhibiting inflammation, the desmoplastic reaction, and the dense stromal remodeling that typically promote PC progression [117,118,119].

A major challenge in evaluating PC risk among T2DM patients is protopathic bias, particularly given that nearly 80% of PC patients present with new-onset diabetes. Since metformin is the gold-standard first-line therapy, it is initiated during the period of highest clinical risk—immediately following T2DM diagnosis. It is probable that some of the observational studies included in this meta-analysis attempted to mitigate this by using new-user designs and incorporating lag-time periods, whereby PC cases diagnosed within the initial months of treatment were excluded. Nevertheless, the superior RR observed with TZDs and DPP4i may be partially influenced by diabetes duration. Patients transitioning to these second-line agents often have longer-standing diabetes. Consequently, the lower risk associated with TZDs and DPP4i may reflect both their specific molecular benefits and a temporal shift away from the initial period of protopathic risk. Several limitations of the present meta-analysis warrant consideration. Firstly, the meta-analysis is constrained by its reliance on observational study data, which precludes the establishment of definitive causality. Secondly, while this meta-analysis provides a comprehensive comparison of metformin against traditional ADs, it is important to note the absence of newer medication classes, such as SGLT2 inhibitors and GLP-1 receptor agonists. Although these agents are now central to the 2026 Standards of Care in Diabetes due to their cardiorenal benefits, our literature search revealed a paucity of studies comparing their specific impact on PC incidence relative to metformin. This represents a critical gap in the current clinical evidence. Thirdly, the included studies exhibited a paucity of granular patient-level data, specifically regarding diabetes duration, cumulative pharmacological exposure, dosage regimens, baseline HbA1c levels, and obesity status. Given that these variables are established modulators of oncogenic risk and may confound the observed associations, the findings of this meta-analysis should be interpreted with prudence. Fourthly, a significant limitation in the current body of literature is the lack of standardized reporting for metformin exposure. Although the anticancer effects mediated via the LKB1/AMPK/mTOR pathway are likely dose-dependent, our meta-analysis was constrained by the inconsistent quantification or total absence of dosage data across the included studies. To address this, we propose that future observational studies adopt a standardized pharmacological reporting framework, incorporating the following three pillars. 1. Quantification by defined daily dose (DDD), adopting the WHO-standardized DDD to facilitate meaningful cross-study comparisons. 2. Cumulative exposure index, reporting the total lifetime dose. 3. Lag-adjusted duration, tracking dosage specifically after a 12-to-24-month “oncological lag-time” to ensure that the drug exposure is associated with true risk reduction rather than the management of subclinical PC symptoms. Implementing such a framework would allow future meta-analyses to carry out meta-regression that could pinpoint the threshold dose required to achieve maximal pancreatic protection.

A critical consideration in interpreting our findings is the role of BMI and baseline adiposity as confounding factors. Obesity is a potent driver of the pancreatic metabolic microenvironment through the induction of chronic low-grade inflammation and oxidative stress [120,121]. As noted in the literature, medications like insulin and TZDs are frequently associated with weight gain, whereas metformin is generally weight-neutral or promotes modest weight loss [122,123]. The absence of BMI-stratified data in the included studies suggests that the “nuanced risk profile” we observed, particularly the lower PC risk associated with metformin compared to insulin, may be partially mediated by these divergent effects on body weight. Clinically, this suggests that for at-risk populations with high adiposity, the selection of a secondary agent should prioritize medications that do not exacerbate weight gain. While our meta-analysis indicates that metformin remains a robust foundation for PC risk reduction, clinical recommendations must account for a patient’s baseline metabolic health; adding agents that improve insulin sensitivity without promoting adiposity may offer a more favorable oncological risk profile than traditional weight-promoting therapies.

The significant statistical heterogeneity observed in Figure 2 and Figure 3A is a common feature of meta-analysis involving large-scale observational data. We have identified several key sources of this variance. Firstly, we identified methodological sources. The included studies utilized diverse health registries, which vary in their coding accuracy for PC and T2DM. Furthermore, the duration of follow-up across studies ranged from 2 to over 10 years. Since PC has a long latency period, shorter studies may capture different risk profiles than longer ones, contributing to variance in the RR. Secondly, we observed biological sources. Heterogeneity is likely driven by differences in patient ethnicity, baseline BMI, and the stage of T2DM progression at the time of metformin initiation. The metformin effect may manifest differently in Asian populations compared to Western cohorts due to variations in genetic risk factors for PC and different lifestyle-related metabolic profiles. Thirdly, while high I^2^ values indicate that the precise pooled RR should be interpreted with caution, they do not necessarily reduce the reliability of the direction of effect. As shown in the forest plots, the majority of large-scale studies consistently favor metformin over SUs and insulin. To address the impact of this variance, we utilized the M-H fixed-effect model to prioritize the influence of high-precision, large-cohort studies. Consequently, our findings remain generalizable as a robust indicator of metformin’s protective trend, even if the exact magnitude of risk reduction varies across different global clinical environments.

## 4. Materials and Methods

### 4.1. Search Strategy and Selection Criteria

This meta-analysis was rigorously conducted in strict accordance with the Preferred Reporting Items for Systematic Reviews and Meta-Analyses (PRISMA) guidelines. The study protocol was prospectively registered in the PROSPERO database (ID: CRD420261342174) to ensure transparency and reproducibility.

A systematic and exhaustive search was executed across several primary electronic databases: PubMed/Medline, Web of Science, Embase, Cochrane Central Register of Controlled Trials (CENTRAL), and ClinicalTrials.gov, encompassing all records published through 2025. This was supplemented by a rigorous search of the gray literature via Google Scholar, including conference proceedings, technical reports, and non-peer-reviewed datasets, to mitigate publication bias. To ensure the inclusion of the most contemporary evidence. The search strategy was re-executed immediately prior to the definitive data synthesis.

The core research question and eligibility criteria were structured using a modified PICOT (Population, Intervention, Comparison, Outcomes, and Type of Study) framework. Inclusion was restricted to studies meeting the following parameters: (P)—patients with T2DM; (I)—involving metformin monotherapy; (C)—against other antidiabetic drugs (ADs), specifically sulfonylureas (SUs), alpha-glucosidase inhibitors (AGIs), thiazolidinediones (TZDs), dipeptidyl peptidase 4 inhibitors (DPP4i), meglitinides as well as insulin; (O)—incidence of PC; and (T)—limited to observational designs (cohort and case–control). The Boolean search string was optimized as follows: ((diabetes) OR (type 2 diabetes) OR (type 2 diabetes mellitus)) AND ((metformin) OR (biguanide)) AND ((type 2 diabetes treatment) OR (type 2 diabetes therapy)) AND ((pancreatic cancer) OR (pancreatic cancer risk)). Exclusion criteria were stringently applied to filter out non-primary research (meta-analysis, review articles, case series, systematic reviews, or case reports), studies with insufficient or non-extractable statistical data, non-English language publications, and retracted articles.

### 4.2. Selection of Studies and Data Extraction

The study selection and data extraction processes were conducted in multiple independent stages by two reviewers concurrently, adhering to rigorous systematic review methodology to minimize bias. The study selection process included three stages. The first stage was initial screening, where reviewers independently screened the titles and abstracts of all articles retrieved from the databases. The second step was duplicate removal, where identified duplicate records were systematically removed from the pool of articles. The third step was the full-text review and inclusion. Each reviewer then assessed the full text of all potentially relevant articles to confirm adherence to the predefined inclusion criteria. To mitigate selection bias, both investigators independently determined whether to reject or include each study. Divergences in inclusion decisions were resolved through discussion until a consensus was achieved.

Following selection, the data extraction process was performed independently by each investigator. Each reviewer populated a separate standardized data collection form with comprehensive information, including: study identifiers (titles, authors, institutions, and registration numbers), methodological details (study design, duration, and publication data), participant characteristics (intervention, number, age, and disease state), and reported outcomes. Subsequently, the data forms were collaboratively reviewed for consistency and accuracy. Discrepancies regarding the classification or interpretation of the results were resolved through negotiation to reach a final consensus. Selected studies were also screened for any missing or ambiguous data.

### 4.3. Statistical Analysis

The extracted data were compiled into a standardized database and subsequently analyzed using the Cochrane Review Manager (RevMan) 5.4 software. The final synthesized results for dichotomous outcomes are reported using the relative risk (RR) metric along with its corresponding 95% confidence interval (CI). The pooled risk ratio (RR) was calculated using the Mantel–Haenszel (M-H) fixed-effect method. The M-H method sums up the weighted experimental events and divides them by the weighted control events across all included studies. The formula is:

$${RR}_{MH}=\frac{\sum \left(\frac{a_{i}\times n_{2i}}{N_{i}}\right)}{\sum \left(\frac{c_{i}\times n_{1i}}{N_{i}}\right)}$$

where a_i_ represents events in the metformin group, c_i_ represents events in the comparative group, n_1i_ represents the total in the metformin group, n_2i_ represents the total in the comparative group, and N_i_ represents the total sample size for that study.

The statistical heterogeneity among study results was quantified using I^2^ statistics and was interpreted as follows: low heterogeneity: I^2^ value of 0–30%; moderate heterogeneity: I^2^ value of 30–60%; and high heterogeneity: I^2^ value over 60%. A *p* < 0.05 was established as the threshold for statistical significance for the overall meta-analysis outcomes. Although statistical heterogeneity (I^2^) was observed in some comparisons, a fixed-effect model was preferred over a random-effects model for several reasons. First, the M-H method is computationally robust and provides better estimates than the inverse variance method when dealing with sparse data or imbalanced group sizes, which are common in large-scale observational cohorts. Second, in meta-analyses involving very large sample sizes (exceeding five million patients in this study), a random-effects model can disproportionately increase the influence of smaller studies with higher variance, potentially biasing the results. By utilizing the fixed-effect model, a conservative estimation of the common treatment effect observed across major registries was prioritized. To ensure the reliability of these findings despite the I^2^ values, rigorous subgroup and sensitivity analyses to confirm that the direction and significance of the RR remained stable across different study populations were conducted. The “Weight” column represents the relative influence of each study on the pooled effect size. Under the M-H fixed-effect model, the weight (W_i_) of each study was calculated by multiplying the number of subjects in the exposed (metformin) group (n_1i_) by the number of events in the control (comparative) group (c_i_), and then dividing by the total sample size (N_i_) of that study (W_i_ = n_1i_ × c_i_/N_i_ for RR). This method effectively prioritizes studies with larger sample sizes and more informative event distributions. Finally, the obtained weight was multiplied by 100% and divided by the sum of all weights. The sum of these weights across all included studies equals 100%.

## 5. Conclusions

**Figure 6:**
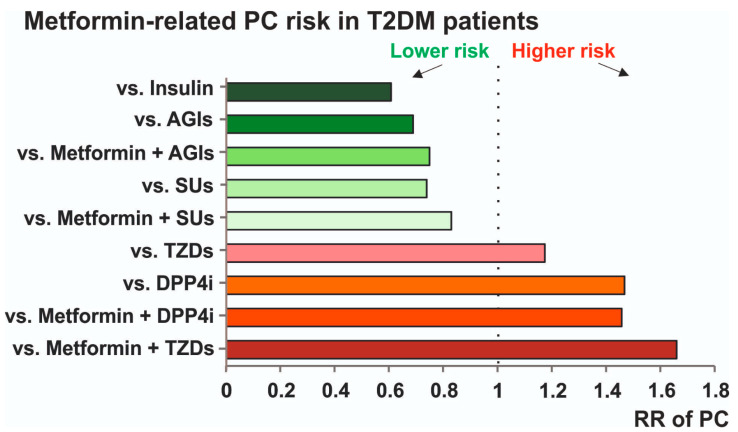
A summary of evidence regarding the risk of PC in T2DM treated with metformin compared to other ADs or metformin combined with other ADs.

Our findings demonstrate that metformin monotherapy users had a lower risk of PC in comparison to users of ADs other than metformin. The deeper analyses comparing metformin monotherapy to specific AD classes revealed a nuanced risk profile. Metformin significantly reduced the risk of PC when compared to monotherapies involving SUs, AGIs, and insulin. Conversely, metformin monotherapy was associated with a markedly elevated PC risk when compared to TZDs and DPP4i. Our results showed that TZD or DPP4i users, both in monotherapy or in combination with metformin, had a lower PC risk than metformin monotherapy users. Figure 6 summarizes the results of our meta-analysis, presenting a metformin-related PC risk in T2DM patients. Taken together, the results of our meta-analysis highlight that users of metformin alone or in combination with TZDs or DPP-4i may benefit more in reducing the risk of PC than those treated with SUs, AGIs, and insulin. These data should be taken into account when the enhancement of antidiabetic treatment is required in patients at risk of PC.

## Data Availability

The original contributions presented in this study are included in the article. Further inquiries can be directed to the corresponding author.
